# Solar-Pumping Upconversion of Interfacial Coordination Nanoparticles

**DOI:** 10.1038/srep41446

**Published:** 2017-01-30

**Authors:** Ayumi Ishii, Miki Hasegawa

**Affiliations:** 1 College of Science and Engineering, Aoyama Gakuin University, 5-10-1 Fuchinobe, Chuo-ku, Sagamihara, Kanagawa, 252-5258, Japan

## Abstract

An interfacial coordination nanoparticle successfully exhibited an upconversion blue emission excited by very low-power light irradiation, such as sunlight. The interfacial complex was composed of Yb ions and indigo dye, which formed a nano-ordered thin shell layer on a Tm_2_O_3_ nanoparticle. At the surface of the Tm_2_O_3_ particle, the indigo dye can be excited by non-laser excitation at 640 nm, following the intramolecular energy transfer from the indigo dye to the Yb ions. Additionally, the excitation energy of the Yb ion was upconverted to the blue emission of the Tm ion at 475 nm. This upconversion blue emission was achieved by excitation with a CW Xe lamp at an excitation power of 0.14 mW/cm^2^, which is significantly lower than the solar irradiation power of 1.4 mW/cm^2^ at 640 ± 5 nm.

Upconversion materials have been recently shed light on their potential to convert low-energy photons into higher-energy photons. The use of upconversion materials is beneficial in applications in the field of light energy, such as lighting, biomarkers, photovoltaics, and photocatalysis. Solar energy is an abundant source of renewable energy, and photoelectric conversion systems have thus been optimized to enable its effective usage. However, the energy conversion efficiency of a single-junction solar cell is limited to the Shockley–Queisser limit of approximately 32% because of its inability to absorb photons with an energy of less than the bandgap of the active materials^1^. Additionally, photovoltaic devices are mainly optimized for outdoor conditions with respect to the air mass (AM) 1.5 spectrum at an illumination intensity of 1 sun (100 mW/cm^2^). However, under cloudy conditions and for use in emerging indoor applications in sensor technology, it is necessary to optimize solar cell performance in different spectral regions, especially in the near-infrared (NIR) region, and at low light intensities between 10^−4^ and 10^−2^ suns. To address these issues, the conversion from low-energy photons to higher-energy photons, called photon upconversion, is a useful technique^2^,^3^,^4^,^5^.

The focus of the present study is the construction of functional interfaces through the complexation of organic and inorganic materials with the aim of developing a novel upconversion emission system that is functional even at low excitation powers, such as sunlight irradiation. The trivalent lanthanide ions (Ln^3+^) were used as the core material for the generation of an efficient upconversion emission. Ln^3+^ shows unique optical properties originating from the electronic transition of the inner-shell 4f orbitals. The emissions of Ln^3+^ appear as sharp bands with long luminescence lifetimes (on the order of micro- or milliseconds) and high chemical/photochemical stability, and they have been used for a wide range of applications in displays, lasers, sensors, electroluminescence and photovoltaic devices^6^,^7^,^8^,^9^,^10^,^11^,^12^. Ln^3+^ has intermediate energy levels and can release emissions from the visible to the near infra-red (NIR) wavelength region. Its excitation energy localized on Ln^3+^ can be transferred to another metal ion via intermetal energy transfer pathways, in which low-energy photons can be converted to higher-energy photons. For instance, the NIR excitation light absorbed by Yb^3+^ as a sensitizer can be converted into visible or ultraviolet light via multiple energy transfers to Er^3+^, Ho^3+^, Nd^3+^ and Tm^3+^, in which the sensitizer (Yb^3+^) and emitters (Er^3+^, Ho^3+^, Nd^3+^ and Tm^3+^) are doped as guests in a dielectric host lattice, such as NaYF_4_^13^,^14^,^15^. Ln-induced upconversion emission results from the electric dipole forbidden (Laporte forbidden) 4f–4f electronic transition with a light absorption coefficient *ε (ε* = 1–100 dm^3^mol^−1^cm^−1^)^16^ that is significantly smaller than that of organic dyes (*ε* = 10^4^–10^5^ dm^3^mol^−1^cm^−1^). Therefore, continuous-wavelength (CW) laser excitation with extremely high incident light intensities (10^3^–10^6^ mW/cm^2^) is required for the upconversion emission of Ln ions^17^.

We succeeded to induce the upconversion emission by a low-power excitation such as sunlight irradiation instead of a laser power source. This system is based on an interfacial Ln complex with an organic dye as a photo antenna on the surface of nanoparticles, in which a low-power excitation is enough to form the upconversion emissive state. It is well known that f–f emissions of Ln^3+^ can be induced by the transfer of energy from an organic compound with a high absorption coefficient, which is accelerated most efficiently by the formation of coordination bonds between Ln^3+^ and an organic ligand. Thus, an organic–inorganic hybrid system with complexation at the interface may realize efficient photoabsorption and energy migration even under low-power light irradiation. Additionally, a novel application of Ln oxide nanoparticles for appropriate upconversion materials was developed in this study. Ln easily reacts with oxygen at ordinary temperature and pressure, and therefore, Ln oxides are the most stable and common Ln compounds^18^. The use of Ln oxide nanoparticles may reduce the difficulty of synthesizing upconversion materials, such as Ln-doped dielectric nanocrystals. In general, the f–f emissions in Ln oxides can easily deactivate by their lattice vibration, and few reports have discussed emissive materials of Ln oxides. For the application of Ln oxides to upconversion materials, it is important to adopt a nano-ordered structure with a large surface area that can react with organic compounds as a photo antenna.

Here, we selected a common indigo dye with coordination site to metal ions as a photo antenna. An indigo dye with a high absorption coefficient in the visible light wavelength region has great potential for use as an energy donor for luminescent Ln ions. It is often used to provide artificial coloration for foods, textiles, cosmetics, drugs, and papers and is also employed to develop new technologies in the medical and electronic fields^19^. The interest in the color of indigo dyes dates back to the eighth century, for example, the ancient Maya and other civilizations in Mesoamerica employed a brilliant blue pigment called Maya blue, which consists of indigo dye stabilized in palygorskite clay^20^. Such nanostructured organic–inorganic hybrid systems with intense colors and high durability have attracted increasing amounts of attention for novel functional materials in the fields of photonics, electronics, magnetics, and catalysis. Furthermore, from the perspective of solar energy use, most researchers have focused on the high absorption properties of indigo dye in the visible light wavelength region^21^.

In an organic–inorganic hybrid nanostructure like the indigo association in Maya blue, the interface is the key to the development of novel functions not possessed by the organic or inorganic component itself. In previous reports^22^,^23^, we found a novel organic–inorganic hybrid system with unique emission properties, which consists of an interfacial complex of Eu and 1,10-phenanthroline at the solid surfaces of the SiO_2_ nanoparticle. In this case, the specific interface condition with complexation can generate the unusual emission of Eu ion. The focus of the current study was the construction of photofunctional interfaces through the complexation of organic and inorganic materials with the goal of enhancing the blue upconversion emission of Tm ions under sunlight irradiation through the fabrication of Yb–indigo dye complexes on core/shell structured nanoparticles.

## Results and Discussion

### Nanoparticle formation and structural analyses

To prepare core/shell structured Tm/Yb oxide nanoparticles, Tm_2_O_3_ nanoparticles with a particle size of approximately 15 nm were immersed in a 10 mM ethanol solution of YbCl_3_ at 70 °C for 60 min. After baking at 400 °C for 60 min, the nano-ordered shell layer of Yb oxides or hydroxides formed on the Tm_2_O_3_ nanoparticles, as evidenced by the lack of observed structural change in the scanning electron microscope (SEM) images (Fig. S1). The elemental compositions of core/shell structured Tm/Yb oxide nanoparticles were confirmed by X-ray photoelectron spectroscopy (XPS) and energy dispersive X-ray spectroscopy (EDS). In the Tm/Yb oxide nanoparticles, Tm and Yb 4d XPS bands were observed at 176 and 185 eV, respectively, which correspond with the bands of each metal oxide (Fig. S2). The EDS results no Cl peak around 2.6 keV (Fig. S3), indicating that Yb oxides or hydroxides formed on the Tm_2_O_3_ nanoparticles through the colloidal suspension and sintering processes.

**Figure 1:**
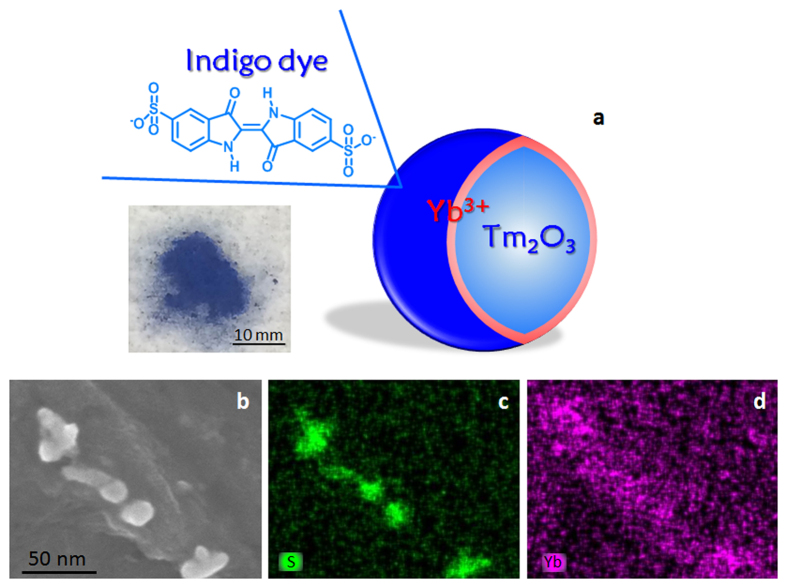
Core/shell structured Tm/Yb oxide nanoparticles coordinated with indigo dye. (**a**) Schematic illustration with molecular components and photograph of the sample. (**b**) SEM image of Tm/Yb oxide nanoparticles coordinated with indigo dyes. (**c**) EDS mapping on S atom. (**d**) EDS mapping on Yb atom.

The Tm/Yb oxide nanoparticles were reacted with an indigo dye (indigo carmine), resulting in bright blue nanoparticles, as shown in Fig. 1a. In the previous works, interfacial complexation with organic dyes and Ln ions has been demonstrated at the solid surface of SiO_2_ or TiO_2_ nanostructure, and has been shown to function as excellent photoemission and photoelectric conversion^22^,^23^,^24^,^25^. Accordingly, the indigo dye coordinated to the Yb ion on the surface of the Tm_2_O_3_ nanoparticles. The results of XPS and Fourier transform infrared spectroscopy (FT-IR) demonstrated the formation of coordination bonds with the metal ion through the carbonyl oxygen and indole nitrogen of the indigo dye on the Tm/Yb oxide nanoparticles (Figs S4 and S5). In the resulting data, the N1s XPS band of the indigo dye at 399.8 eV was partially shifted to 400.5 eV on the higher energy side by coordinating to the Tm/Yb oxide nanoparticles. Furthermore, the stretching vibration of the C=O bond of the indigo dye near 1700 cm^−1^ disappeared from the Tm/Yb oxide nanoparticles, followed by evidence of bonding between the Yb cation and the dye through oxygen. This was supported by the appearance of a new peak at 602 cm^−1^ assigned to the Yb–O bonding. The N–H stretching band of the indigo dye at 3360 cm^−1^, which relates the coordination bond, was also significantly weakened. These data indicate that the indigo dye coordinated to the Yb ions on the Tm_2_O_3_ nanoparticles with bidentate formation through nitrogen and oxygen, which is similar to the organic–inorganic complex structure of Maya blue associated with the indigo bound to a surface Al ion of palygorskite clay^26^,^27^.

Figure 1b shows an SEM image of Tm/Yb oxide nanoparticles coordinated with the indigo dye. Compared with the Tm_2_O_3_ and Tm/Yb oxide nanoparticles shown in Fig. S1, the average Tm_2_O_3_ particle size of 15 nm was almost unchanged by the coordination of indigo dye on the surface, indicating that the Yb–indigo dye interfacial complex formed a nano-ordered thin shell layer on the nanoparticle. The indigo dye on the Tm/Yb nanoparticles appeared to form a smooth surface with close packing between the particles. The EDS mappings in Fig. 1c and d indicate that the Yb ions of the shell layer and the S atoms of the indigo dye were uniformly distributed on nanoparticles, and the dye was thus uniformly coordinated with Yb ions at the surface of the nanoparticles.

**Figure 2:**
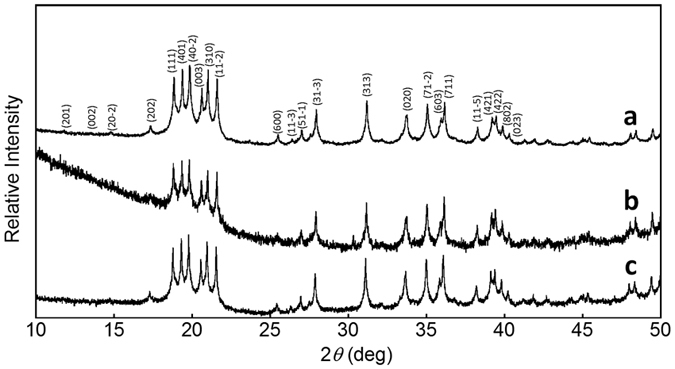
Synchrotron XRPD patterns (*λ* = 0.9988 Å). (**a**) Tm_2_O_3_ nanoparticle, (**b**) Tm/Yb oxide nanoparticle, and (**c**) Tm/Yb oxide nanoparticle coordinated with indigo dye.

The synchrotron X-ray powder diffraction (XRPD) patterns are shown in Fig. 2. The cell parameters of each compound are summarized in Table S1. Structural analysis of Tm_2_O_3_ nanoparticle yielded a monoclinic structure (space group *C*_2/m_) with *a* = 13.802 Å, *b* = 3.441 Å, *c* = 8.506 Å, *β* = 100.175°, *V* = 397.66 Å^3^, and *Z* = 6 (Table S1 and Fig. S6). As shown in Fig. S7, each Tm ion was surrounded by six O atoms, forming a distorted octahedral structure. The nanoparticle showed a lower crystal system than the bulk Tm_2_O_3_ powder with a cubic structure^28^ because of the large number of low-coordination Tm ions existing at the surface of nanoparticles with a large specific surface area^29^. Such low symmetrical conditions in the nanoparticle allow the occurrence of the f–f transition of Ln ions, which is basically forbidden, thus strengthening the emission intensity.

The crystal size *D* of Tm_2_O_3_ was estimated to be approximately 16 nm using the Scherrer equation, *D* = 0.9*λ*/*β* cos *θ*^30^, where *λ* is the X-ray wavelength and *β* is the full width at half-maximum (FWHM, given in radians) of the diffraction peak at the Bragg angle *θ*. Slight crystal growth was observed on the particle with Yb ions (*D *≈ 18 nm) and indigo dye (*D* ≈ 25 nm). These results support the SEM observation of close packing in indigo dye-coordinated Tm/Yb nanoparticles. Additionally, the cell volume of the Tm_2_O_3_ nanoparticles increased after they had been coated with Yb ions (*V* = 398.73 Å^3^) and indigo dye (*V* = 401.44 Å^3^), although the crystal structure was maintained. This may indicate that the surface conditions of the nanoparticles affect the crystal growth and bond length between Tm and O atoms.

### Multi-step energy transfer

**Figure 3:**
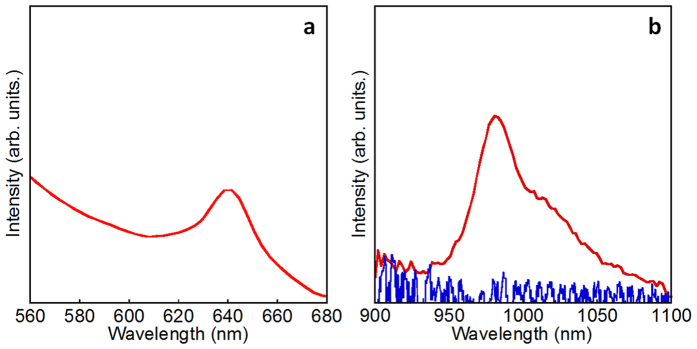
Energy transfer from indigo dye to Yb^3+^at the surface of core/shell oxide nanoparticles. (**a**) Excitation spectrum of Yb/Yb oxide nanoparticles coordinated with indigo dyes monitored at 987 nm. (**b**) Emission spectra of Yb^3+^ in Yb/Yb oxide nanoparticles coordinated with indigo dye (red line) and Tm/Yb oxide nanoparticle coordinated with indigo dye (blue line). The excitation wavelength is 640 nm.

To clarify the transfer of energy from indigo dye to Yb ions at the surface of nanoparticles, Yb_2_O_3_ nanoparticles were used as the core structure and coated with a shell composed of Yb–indigo dye interfacial complexes. The observed luminescence spectrum is shown in Fig. 3. The Yb/Yb oxide nanoparticles with indigo dye exhibited an emission band at 987 nm, which was assigned to the ^2^F_5/2_ → ^2^F_7/2_ transition of the Yb ions. The excitation spectrum monitored at the emission band position corresponds to the π−π* transition of the indigo dye (Fig. S8)^31^,^32^, confirming that energy was transferred from the indigo dye to the Yb ion at the surface of the nanoparticles. The interfacial energy transfer process appeared to be efficient because the fluorescence band of the indigo dye, shown in Fig. S8, was eliminated by the complexation to Yb ion at the interface. It should be pointed out, for comparison, the NIR emission of Yb ions by the 640 nm excitation was not present in Tm/Yb oxide nanoparticles coordinated with indigo dye, as shown in Fig. 3b. After the energy transfer from the indigo dye to the Yb ions, the excitation energy could then be transferred to Tm ions at the interface.

**Figure 4:**
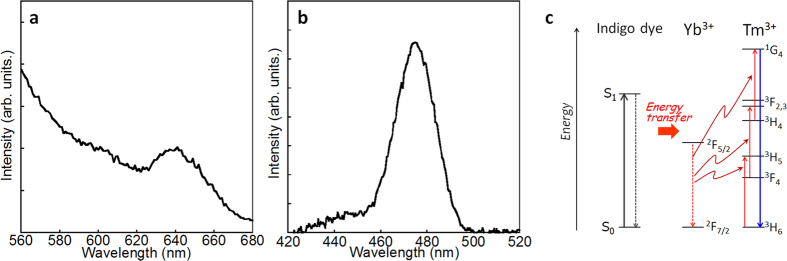
Upconversion emission of Tm/Yb oxide nanoparticles induced by the coordination of indigo dye. (**a**) Excitation spectrum monitored at 475 nm. (**b**) Emission spectrum measured by excitation using a CW Xe lamp at 640 nm. (**c**) Schematic of the energy migration pathway.

Figure 4b shows the luminescence spectrum of the Tm/Yb oxide nanoparticles coordinated with indigo dye in the visible wavelength region. Under excitation at 640 nm, the nanoparticles generated a blue emission at 475 nm. This blue emission originated from the ^1^G_4_ → ^3^H_6_ transition of the Tm ions and may have been induced by red light excitation. In the case of a Tm_2_O_3_ nanoparticle coated with Tm ion and coordinated with the dye, the 475 nm emission was never observed (Fig. S9), indicating that the energy transfer efficiency from the dye to Tm ion seems to be quite low and the direct energy transfer from the dye to Tm ion hardly occurs. That is, this blue emission corresponds to an upconversion emission following a multi-step energy transfer process from the dye to Yb and subsequently from Yb^3+^ to Tm^3+^ at the interfacial complex. The excitation spectrum monitored at 475 nm supports the conclusion that the upconversion emission occurred through the energy transfer from the π−π* absorption of the indigo dye at approximately 640 nm (Fig. 4a). The upconversion emission of Tm^3+^ in the oxide compound without the coordination of indigo dye was not observed following the direct excitation of Yb^3+^. The excited state of Yb^3+^ as an energy donor for Tm^3+^ was efficiently formed by the energy transfer from the indigo dye, not by the direct excitation of the forbidden f–f transition (Fig. 4c).

### Excitation by low-power irradiation

**Figure 5:**
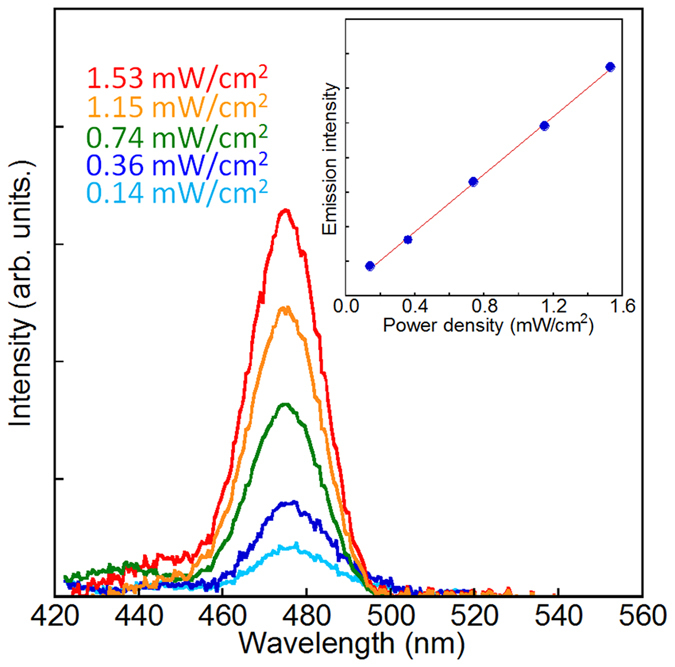
Dependence of the upconversion emission on the excitation power density. Emission spectra of Tm/Yb oxide nanoparticles coordinated with indigo dye measured at different power densities using a CW Xe lamp with an excitation wavelength of 640 nm. The inset shows the direct proportional relationship between the excitation power density and upconversion emission intensity.

The emission intensity is dependent on the excitation power, as shown in Fig. 5. It is remarkable that this upconversion blue emission could be obtained through excitation by a CW Xe lamp at a significantly lower power than the solar irradiation power of 1.4 mW/cm^2^ at 640 ± 5 nm^33^. The upconversion emission was still observed even at an excitation power as low as 0.14 mW/cm^2^. In an upconversion system of Ln ions in which sequential absorption occurs via intermediate excitation states, the number of photons required to populate the upper emitting state under unsaturated condition is generally given by^34^


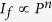


where *I*_f_ is the upconversion emission intensity, *P* is the excitation light power, and *n* is the number of photons required for upconversion. To induce luminescence from the ^1^G_4_ state of Tm^3+^ by upconversion via the lower energy levels, three photons are needed (*n* = 3), as shown in Fig. 4c. However, in the present interfacial complex system, a direct proportional relationship between *I*_f_ and *P* was obtained (*n* = 1), indicating that the large population of the excited state of Yb^3+^ generated by the energy transfer from the dye may enable the effective intermetal energy transfer from Yb^3+^ to Tm^3+^ at the interface.

The absolute quantum yield *ϕ*_UC_ of the upconversion emission was measured using an integrating sphere and a 150 W Xe lamp with a low power density (*ca.* 0.01 mW/cm^2^) at 640 nm. Remarkably, the *ϕ*_UC_ of Tm/Yb oxide nanoparticles coordinated with indigo dye reached 0.3% even at such low-power light irradiation. To the best knowledge of the present authors, this is the first observation of Ln^3+^ -based upconversion emission by extremely low-power excitation, such as solar light irradiation, which is induced via an effective energy migration pathway, consisting of intramolecular transfer from the dye to Yb^3+^ followed by intermetal transfer from Yb^3+^ to Tm^3+^, at the interface of the organic–inorganic hybrid nanoparticles.

## Conclusion

In conclusion, we successfully developed a novel upconversion emission system composed of core/shell structured Yb/Tm oxide nanoparticles coordinated with indigo dyes. This system achieved upconversion blue emissions even at a low-power light excitation, such as sunlight irradiation, which can be only induced by formation of the interfacial complex on the nanoparticles. A Yb–indigo dye interfacial complex was formed as a nano-ordered thin shell layer on Tm_2_O_3_ nanoparticles. At the surface of Tm_2_O_3_, intermolecular energy is efficiently transferred from the indigo dye to the Yb ion, following the upconversion of the excitation energy on the Yb ion to the blue emission of the Tm ion. This upconversion blue emission could be obtained at an excitation power significantly less than that of solar irradiation, and such a high efficient system based on the Ln^3+^ emission under low excitation power has not been reported until now. The phenomenon exhibited in the nanoparticles investigated in this study may be applied to future photo-energy conversion systems. An investigation into how to increase the photon upconversion intensity by suppressing the self-quenching process between Tm ions in core oxide nanoparticles is currently in progress.

## Methods

### Sample preparation

Tm_2_O_3_ nanoparticles with a diameter of approximately 15 nm (Kanto Chemical Co., Inc.) were sintered at 200 °C for 20 min in the presence of nitrogen to remove the water absorbed at the surface. The oxide nanoparticles (10 wt%) were immersed in a 10 mM ethanol solution of YbCl_3_ ∙ 6H_2_O (Kanto Chemical Co., Inc.) and stirred at 70 °C for 60 min. After filtration, the nanoparticles were gradually baked from 110 to 400 °C, and kept at 400 °C for 60 min. The indigo dye (indigo carmine, Tokyo Chemical Industry Co., Ltd.) was dissolved into water (1.25 mM) and kept at pH 8 with a small amount of triethylamine. Tm/Yb oxide nanoparticles (10 wt%) were reacted with the water solution of indigo dye. The colloidal suspension was stirred for 60 min. After filtration, the resulting blue nanoparticles were rinsed with ethanol and dried under vacuum. Yb/Yb and Tm/Tm oxide nanoparticles were also prepared in the same way using Yb_2_O_3_ nanoparticles and TmCl_3_ ∙ 6H_2_O (Kanto Chemical Co., Inc.).

### Apparatus

SEM images were obtained using an Ultra-55 microscope (Carl Zeiss AG) equipped with a secondary in-lens electron detector, together with a QUANTAX detector (Bruker Corporation) for EDS. XPS was performed using an AXIS Ultra delay-line detector (DLD) (Kratos Analytical) equipped with a monochromatic Al-Kα X-ray source (1253.6 eV); the binding energies were calibrated at the Au 4 f level (84.0 eV). Synchrotron X-ray powder diffraction (XRPD) patterns were obtained with a large Debye–Scherrer camera installed at the BL02B2 beamline (SPring-8), using an imaging plate as the detector and an incident X-ray wavelength of 0.9988 Å. FTIR spectra were measured in attenuated total reflection (ATR) mode with a diamond crystal using the Nicolet iS5 infrared spectrometer (Thermo Scientific). Electronic absorption and luminescence spectra were recorded on a UV-3100 spectrophotometer (Shimadzu Corporation) with an absolute specular reflectance attachment and a Jobin Yvon FluoroLog 3–22 (Horiba Scientific), respectively. The excitation light intensity was adjusted using neutral-density (ND) filters. Fluorescence quantum yields were measured using a C9920-02 Absolute PL Quantum Yield Measurement System (Hamamatsu Photonics K.K.).

## Additional Information

**How to cite this article**: Ishii, A. and Hasegawa, M. Solar-Pumping Upconversion of Interfacial Coordination Nanoparticles. *Sci. Rep.* **7**, 41446; doi: 10.1038/srep41446 (2017).

**Publisher's note:** Springer Nature remains neutral with regard to jurisdictional claims in published maps and institutional affiliations.

## Supplementary Material

Supplementary Information
