# Supplementary material for: Solar-Pumping Upconversion of Interfacial Coordination Nanoparticles
Source: Sci Rep. 2017 Jan 30;7:41446. doi: 10.1038/srep41446 (PMC5278413; doi:10.1038/srep41446)
Supplement: Supplementary Information [file srep41446-s1.pdf]

Supplementary information

Solar-Pumping Upconversion of Interfacial Coordination Nanoparticles

Ayumi Ishii, and Miki Hasegawa

College of Science and Engineering, Aoyama Gakuin University, 5-10-1 Fuchinobe, Chuo-ku, Sagamihara, Kanagawa, 252-5258, Japan
E-mail: ayumi@chem.aoyama.ac.jp (A.I.); hasemiki@chem.aoyama.ac.jp (M.H.)

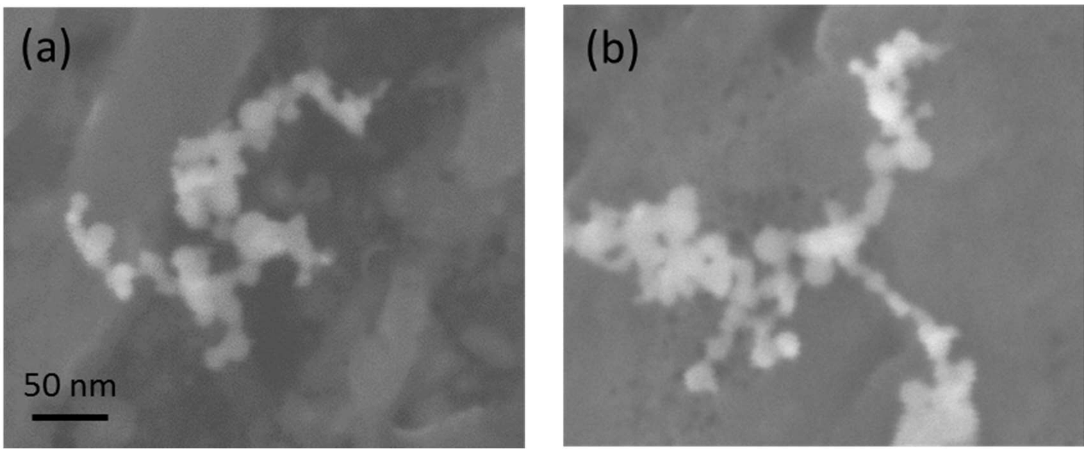

Figure S1. SEM images of (a) Tm_2O_3 and (b) core/shell structured Tm/Yb oxide nanoparticles.

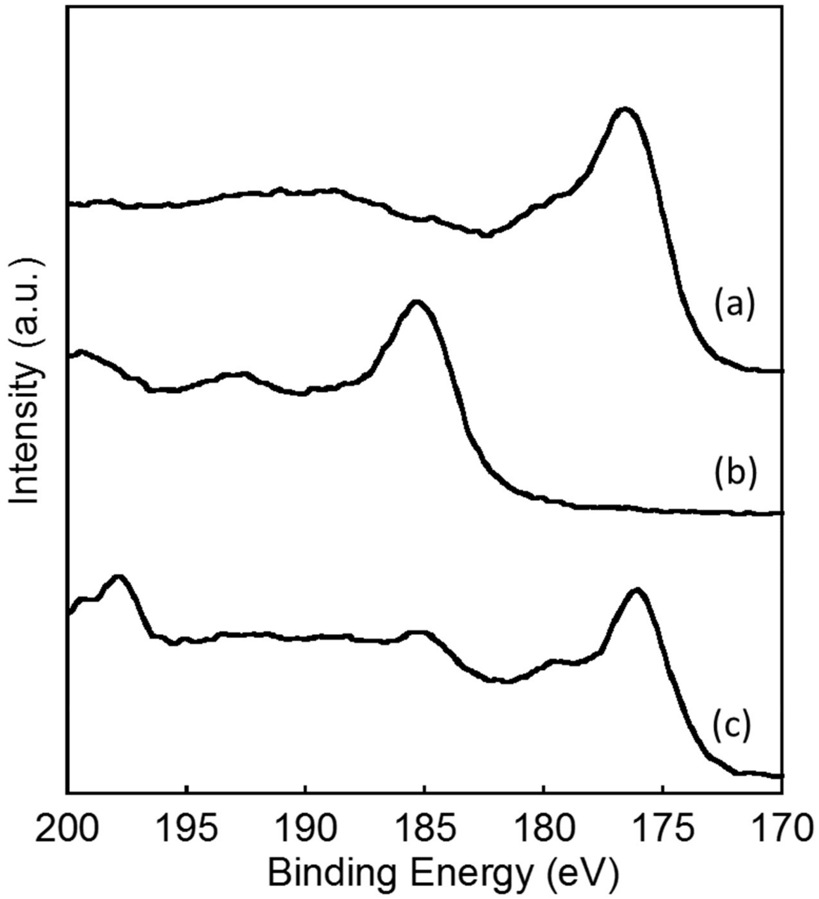

Figure S2. Tm 4d and Yb 4d XPS bands of (a) Tm_2O_3, (b) Yb_2O_3 and (c) Tm/Yb oxide nanoparticle.

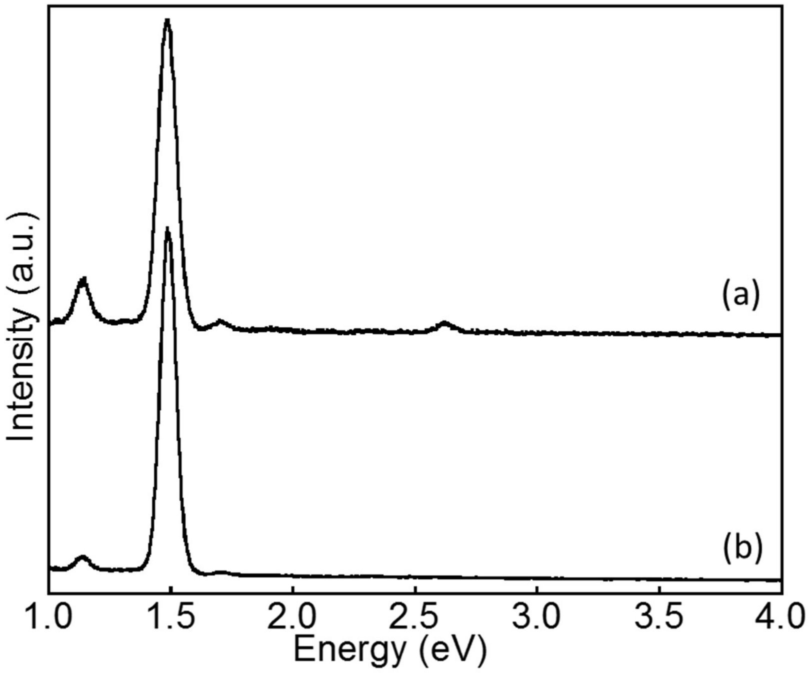

Figure S3. EDS patterns of core/shell structured Tm/Yb oxide nanoparticles (a) as prepared and (b) after sintering.

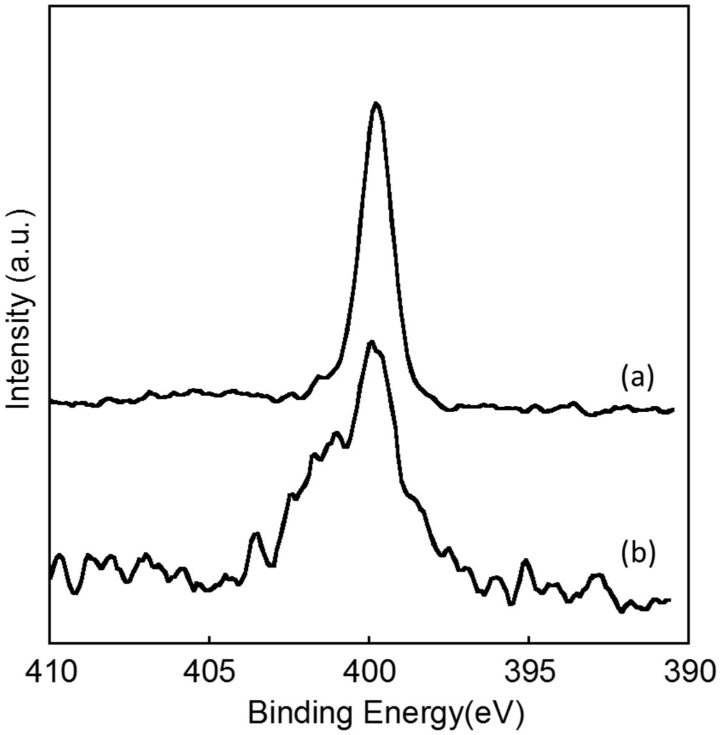

Figure S4. N 1s XPS bands of indigo dye (a) itself and (b) on the Tm/Yb oxide nanoparticles.

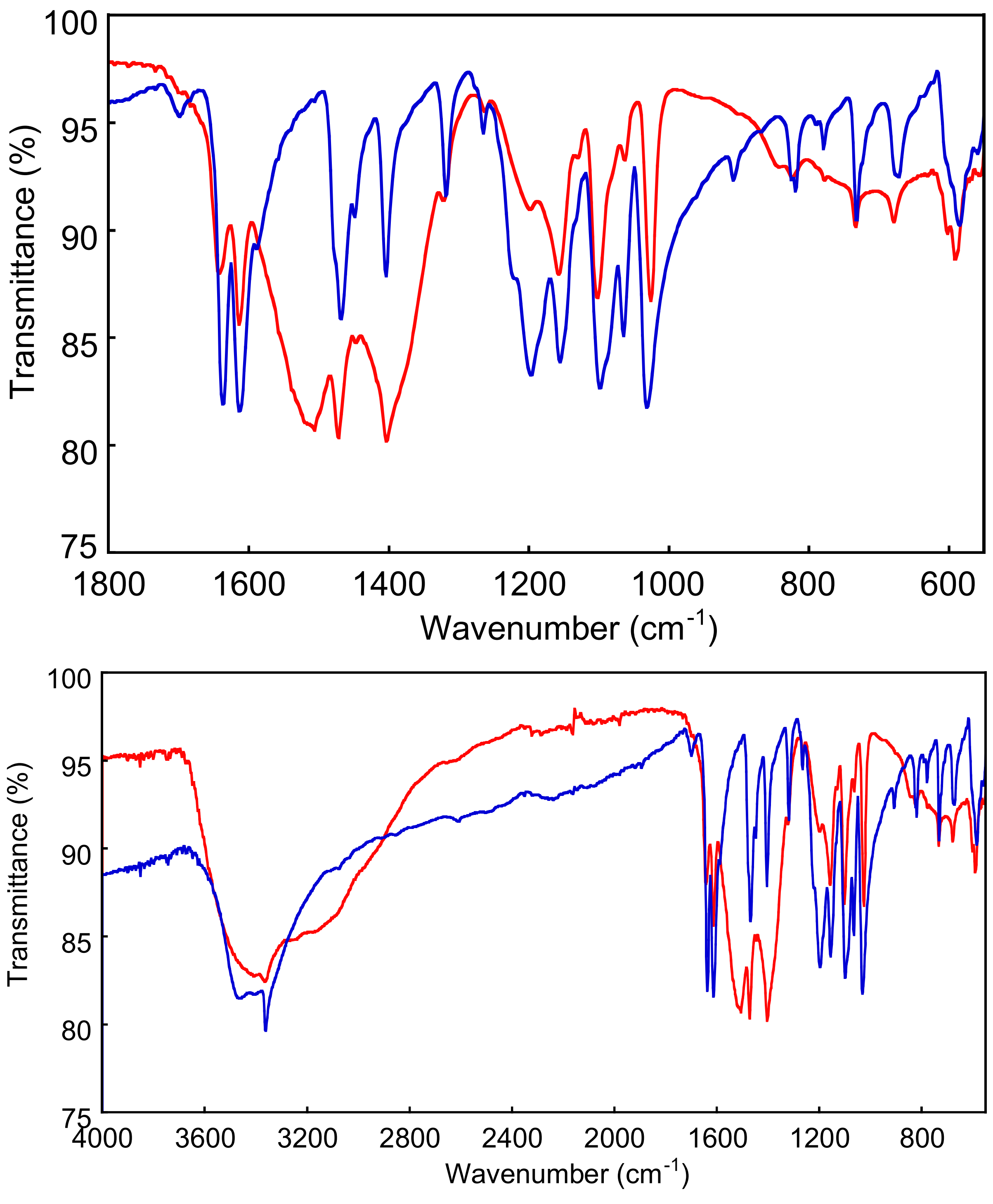

Figure S5. FT-IR spectra of indigo dye itself (blue line) and on the Tm/Yb oxide nanoparticles (red line) (top: 400-1800 cm^{-1}, bottom: 400-4000cm^{-1}).

Table S1. The cell parameters of nanoparticles.

	Tm_2O_3 nanoparticle	Tm/Yb oxide nanoparticle	Indigo dye coordinted Tm/Yb oxide nanoparticle
Crystal system	Monoclinic	Monoclinic	Monoclinic
Space group	$C_{2/m}$	$C_{2/m}$	$C_{2/m}$
a (Å)	13.802	13.812	13.845
b (Å)	3.441	3.443	3.452
c (Å)	8.506	8.517	8.535
β (°)	100.175	100.174	100.203
V (Å^3)	397.66	398.73	401.44
Z	6	6	6

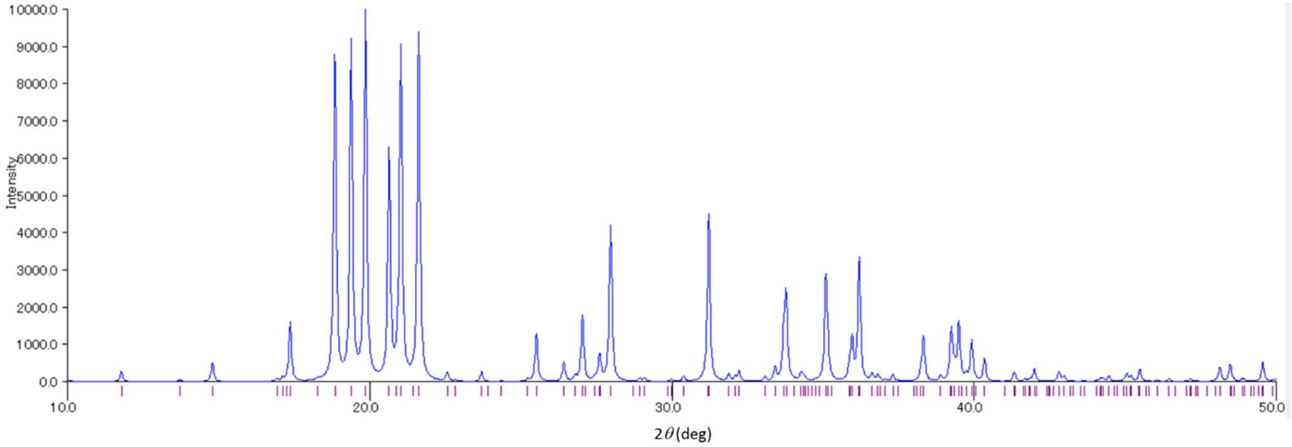

Figure S6. A simulated XRD pattern of Tm_2O_3 nanoparticle. The refinement parameters R_{wp} and R_I were estimated as 0.13 and 0.17, respectively.

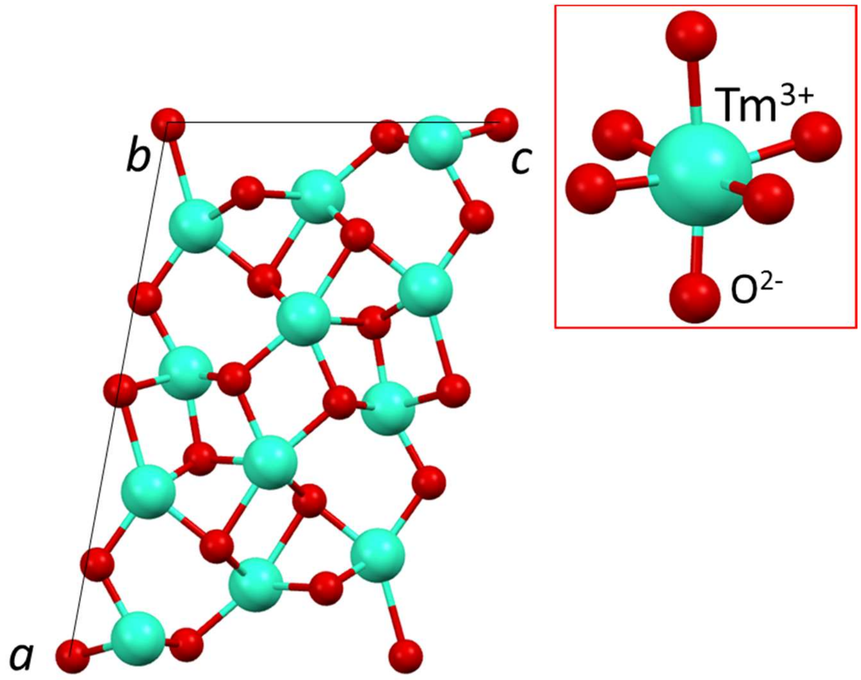

Figure S7. Crystal structure along b axis. The crystal structure of Tm_2O_3 nanoparticle was determined by Rietveld refinement of the XRPD data. Inset shows a coordination geometry around Tm^{3+}.

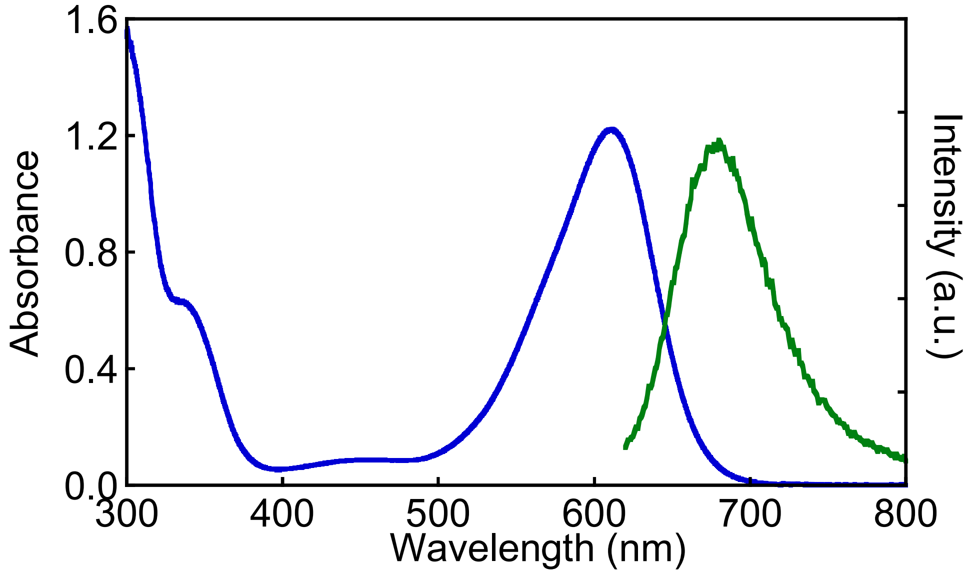

Figure S8. Absorption (blue line) and fluorescence (green line, λ_{ex} = 610 nm) spectra of indigo dye in water.

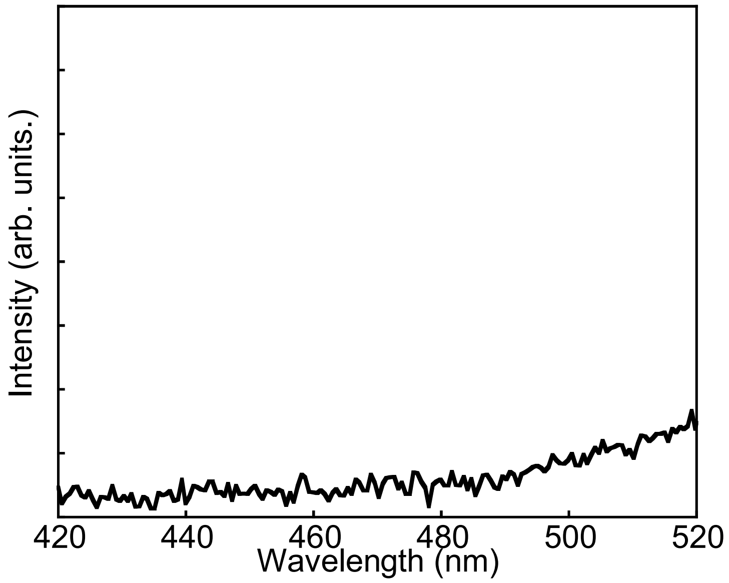

Figure S9. Emission spectra of Tm^{3+} in Tm/Tm oxide nanoparticles coordinated with indigo dye measured by excitation using a CW Xe lamp at 640 nm.
